# Metabolomics and Sensory Evaluation Reveal the Aroma and Taste Profile of Northern Guangdong Black Tea

**DOI:** 10.3390/foods14142466

**Published:** 2025-07-14

**Authors:** Jialin Chen, Binghong Liu, Yide Zhou, Jiahao Chen, Yanchun Zheng, Hui Meng, Xindong Tan, Peng Zheng, Binmei Sun, Hongbo Zhao, Shaoqun Liu

**Affiliations:** 1 College of Horticulture, South China Agricultural University, Guangzhou 510642, China; chenjialin43@126.com (J.C.); arthurlauuu@foxmail.com (B.L.); zhengp@scau.edu.cn (P.Z.); binmei@scau.edu.cn (B.S.); zhao@scau.edu.cn (H.Z.); 2 Goldsands Tea Research Institute, South China Agricultural University, Guangzhou 510642, China

**Keywords:** black tea, HPLC-UV, GC-MS, northern Guangdong black tea

## Abstract

The sensory quality of black tea is intrinsically linked to cultivar genetics, yet comprehensive characterization of flavor compounds in emerging northern Guangdong black tea (NGBT) remains limited. This study employed high-performance liquid chromatography-ultraviolet (HPLC-UV) and headspace solid-phase microextraction coupled with GC-MS (HS-SPME-GC-MS) to analyze non-volatile and volatile compounds in five NGBT cultivars—Jinshahong (JSH), Danxia No.1 (DXY), Danxia No.2 (DXE), Yingde Black Tea (QTZ), and Yinghong No.9 (YHJ)—alongside sensory evaluation. Orthogonal partial least squares-discriminant analysis (OPLS-DA) identified key non-volatile discriminants (VIP > 1) ranked by contribution: total catechins > simple catechins > CG > EGCG > ester catechins > EGC. HS-SPME-GC-MS detected 97 volatiles, with eight aroma-active compounds exhibiting OAV > 1 and VIP > 1: Geraniol > Methyl salicylate > Linalool > *β*-Myrcene > Benzyl alcohol > (*Z*)-Linalool Oxide > Phenethyl alcohol > (*Z*)-Jasmone. These compounds drive cultivar-specific aromas in NGBTs. Findings establish a theoretical framework for evaluating cultivar-driven flavor quality and provide novel insights for targeted breeding and processing optimization of NGBTs.

## 1. Introduction

Black tea, recognized as the world’s most consumed tea category, is globally prized for its distinctive organoleptic properties [1,2]. Yingde City and Renhua County—situated within Shaoguan Prefecture in northern Guangdong—have been identified as Guangdong’s earliest tea cultivation zones, possessing a profound tea-cultural heritage [3,4]. This region’s unique terroir, characterized by Danxia landforms, karst topography, and subtropical climate [5,6], is acknowledged for fostering exceptional tea germplasm diversity [7,8].

Although tea quality is theoretically influenced by multiple factors like cultivar, tree age, processing, nutrition, and post-harvest management, cultivar is established as a primary determinant of sensory characteristics. While GC-MS has been extensively employed to characterize aroma-active compounds like terpenes and esters contributing to floral/honey notes [9,10,11,12], and HPLC-UV routinely quantifies non-volatile taste components like catechins, amino acids, and gallic acid contributing sensory attributes like briskness [13,14], existing research has predominantly focused on single-cultivar process optimization or static compound-class analyses. Consequently, mechanistic gaps persist in understanding key sensory differentiators among northern Guangdong black tea (NGBT) cultivars.

To address these gaps, five representative NGBT cultivars were selected for investigation: Yinghong No. 9 (YHJ), a clonal large-leaf variety systematically bred from Yunnan large-leaf germplasm in 1961 with significant commercial presence [15]; Yingde Black Tea (QTZ), a historically important open-pollinated landrace [16]; Danxia No. 1 (DXY) and Danxia No. 2 (DXE), high-aroma cultivars developed from wild variants of Renhua white-haired tea [17,18]; and Jinshahong tea (JSH), an emerging premium selection derived from Renhua Danxia tea populations [19]. These cultivars are cultivated under comparable climatic conditions, latitudes, longitudes, and annual precipitation. While cultivar-mediated metabolite regulation is documented to shape flavor profiles [20], characteristic compounds distinguishing these NGBT cultivars—especially emerging types like JSH—remain inadequately defined, hindering targeted utilization.

This study was therefore designed to integrate GC-MS, HPLC-UV, and sensory evaluation for systematic elucidation of volatile/non-volatile compositional differences among JSH, DXY, DXE, QTZ, and YHJ, establishing predictive “chemical composition-sensory attribute” correlation models. Findings are expected to provide scientific foundations for NGBT germplasm evaluation while supporting breeding programs and processing optimization for high-aroma black teas, addressing critical knowledge gaps in multi-cultivar flavor chemistry.

## 2. Materials and Methods

### 2.1. Tea Samples

**Figure 1:**
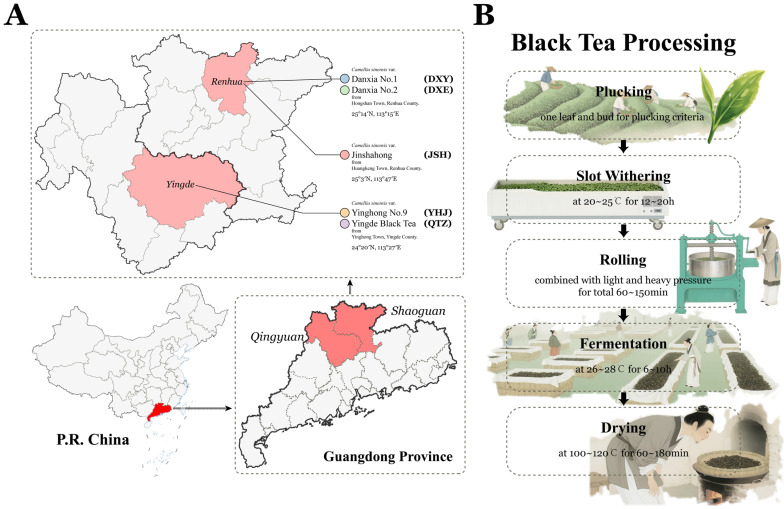
Sampling location (**A**) and processing (**B**) of 5 NGBTs.

Jinshahong (JSH) was provided by Guangdong Goldsands Tea Co., Ltd. (Guangdong, China). Danxia No.1 (DXY), Danxia No.2 (DXE), Yinghong No.9 (YHJ), and Yingde Black Tea (QTZ) were provided by Guangdong Xinxi Tea Co., Ltd. (Guangdong, China). All the tea samples were plucked as one leaf and bud criteria in the spring of 2025 and processed as following steps: plucking (one leaf and bud) → slot withering (20~25 °C, 12~20 h) → rolling (combined with light and heavy pressure for total 60~150min) → fermentation (26~28 °C, 6~10 h) → drying (100~120 °C, 60~180 min) (Figure 1).

### 2.2. Chemical Reagents

Alkane standard solution (C_9_–C_21_) for calculating linear retention indices (RIs) was supplied by TanMo Quality Testing Technology Co., Ltd. (Beijing, China). The internal standard solution was prepared using dichloromethane prior to use [21]. Ultrapure water was produced using a Barnstead GenPure Pro system (Thermo Fisher Scientific, Waltham, MA, USA). The following reference standards were acquired: ethyl decanoate, theanine, and a series of catechins comprising catechin (C), epicatechin (EC), epigallocatechin (EGC), epicatechin gallate (ECG), gallocatechin gallate (GCG), and epigallocatechin gallate (EGCG); all were sourced from Shanghai Yuanye Biotechnology Co., Ltd. (Shanghai, China). Additionally, the caffeine standard was obtained from Beijing Weiyesi Metrology Technology Research Institute (Beijing, China).

### 2.3. High-Performance Liquid Chromatography–Ultraviolet (HPLC-UV) Analysis of Caffeine, Theanine, and Catechins

Caffeine, theanine, and catechin content were quantified using high-performance liquid chromatography–ultraviolet according to the method in our previous research [22,23] (HPLC; Waters Alliance 2695 system equipped with a 2489 UV/Vis detector, Waters Technologies, Milford, MA, USA). Analytes were identified by comparing retention times to authentic standards and quantified using calibration curves (Table S2). All analyses were performed in triplicate.

#### 2.3.1. Sample Preparation for the Analysis of Caffeine, Theanine, and Catechins

For caffeine extraction, tea powder (0.1 g) and magnesium oxide (0.45 g) were combined in a 50 mL centrifuge tube, soaked in 30 mL of 100 °C ultrapure water for 30 min, and ultrasonicated for 30 min. The mixture was then centrifuged (10,000 rpm, 3 min) and the supernatant filtered through a 0.22 µm membrane (Millipore, Jinteng Experimental Equipment Co., Ltd., Tianjin, China).

For theanine extraction, tea powder (0.1 g) in a 10 mL centrifuge tube was soaked in 10 mL of 100 °C ultrapure water for 30 min, centrifuged (6000 rpm, 10 min), and the supernatant filtered (0.22 µm membrane, Millipore, Jinteng).

For catechins extraction, tea powder (0.2 g) in a 10 mL centrifuge tube was extracted with 8 mL of 70% methanol (in ultrapure water) via ultrasonic water bath for 30 min. After centrifugation (10,000 rpm, 3 min), the supernatant was filtered (0.22 µm membrane, Millipore, Jinteng).

#### 2.3.2. HPLC-UV Analysis of Caffeine, Theanine, and Catechins

For caffeine analysis, the filtrate (10 µL) was injected onto an XSelect HSS C18 SB column (4.6 × 250 mm, 5 µm; Waters) maintained at 25 ± 1 °C. Isocratic elution was performed at 0.9 mL/min using methanol (A) and ultrapure water (B) as follows: 45% A and 55% B (0–14 min). Detection occurred at 280 nm in the UV detector.

For theanine analysis, the filtrate (10 µL) was injected into an RP-C18 column (250 mm × 4.0 mm, 5 µm; Waters) at 35 ± 1 °C. The specific HPLC procedure was based on the method of Mei et al. [24]. The isocratic elution proceeded at 0.5 mL/min using 100% ultrapure water (A) and 100% acetonitrile (B) as follows: initial 100% B (0–12 min), decreased linearly to 20% B (12–14 min), maintain level for 5 min (14–19 min), then increased linearly to 100% B (19–21 min), and maintain level for 4 min (21–25 min). Detection was set to 210 nm in the UV detector.

For catechin analysis, the filtrate (10 µL) was injected onto an XSelect HSS C18 SB column (4.6 × 250 mm, 5 µm; Waters) at 25 ± 1 °C. The gradient elution (0.9 mL/min) employed 0.1% formic acid in water (A) and 100% acetonitrile (B) as follows: initial 8% B (0–5 min), increased linearly to 25% B (5–14 min), and then decreased linearly to 8% B (14–30 min). Detection occurred at 280 nm in the UV detector.

### 2.4. Gas Chromatography–Mass Spectrometry (GC-MS) Analysis of Volatile Compounds

Volatile compounds were extracted using headspace solid-phase microextraction (HS-SPME) as described in Chen et al. [21]. For HS-SPME, 2 g of tea powder was loaded into a 40 mL headspace vial. Each sample received 5 mL of saturated NaCl solution, 10 μL of internal standard solution B (1 μL ethyl decanoate (0.864 g/mL) was added in 999 μL dichloromethane as solution A, and then 10 μL of solution A was added into 990 μL dichloromethane as solution B), followed by sealing with aluminum caps. After equilibration (80 °C, 15 min) in a metal bath, volatiles were adsorbed onto a DVB/CAR/PDMS fiber (50/30 μm, 2 cm) at 80 °C for 40 min. The fiber was then thermally desorbed in the GC inlet at 250 °C for 3 min.

Analysis used an Agilent 1890B GC coupled to a 5977A MS. Separation occurred on an HP-5MS capillary column (30 m × 0.25 mm × 0.25 μm) with helium carrier gas (1.0 mL/min, splitless mode). The oven program: 50 °C (hold 1 min), ramp at 5 °C/min to 220 °C (hold 5 min). MS settings: electron ionization (70 eV), ion source 230 °C, scan range *m/z* 30–400, solvent delay 4 min. All samples were analyzed in triplicate.

### 2.5. Data Analysis of GC-MS

Volatiles were identified by matching the retention index (RI) and mass spectra to reference databases [25]. RI values were calculated from C_9_–C_21_ n-alkanes analyzed under identical conditions using the following equation:

(1) $$RI=100n+100\times \frac{RT\left(x\right)-RT\left(n\right)}{RT\left(n+1\right)-RT\left(n\right)}$$

where *RT*(*x*), *RT*(*n*), and *RT*(*n* + 1) represent the retention times (min) of the target compound, n-alkane, and (*n* + 1)-alkane, respectively. Compounds were considered identified if their RI deviated by <15 units from the NIST14 database and their spectral match factor exceeded 90/100.

Volatile compound concentrations were calculated using the internal standard method:

(2) $$C_{i}=\frac{S_{i}}{S_{is}}\times \frac{m_{is}}{m}$$

where *C_i_* is concentration of volatile compound *i* (μg/kg), *S_i_* is the peak area of compound *i*, *S_is_* is the peak area of the internal standard, *m* is the mass in g of sample, and *m_is_* is the mass in ng of the internal standard.

### 2.6. Calculation of the Odor Activity Value (OAV)

The odor activity value (OAV) for each volatile compound was calculated by dividing its concentration by its odor threshold in water, which is widely applied to assess the contribution of compounds to the tea aroma [26]. An OAV magnitude indicates the compound’s contribution to the tea’s flavor profile: values below 1 (0 ≤ OAV < 1) suggest that the odorant is not detectable by the human nose, while values of 1 or higher (OAV ≥ 1) signify a significant contribution to the overall aroma [27]. The calculation is expressed by the formula:

(3) $$OAV=\frac{C_{i}}{T_{i}}$$

where *C_i_* is the concentration of compound *i* (μg/kg), and *T_i_* is the odor threshold (OT) of compound *i* (μg/kg).

### 2.7. Sensory Evaluation

Sensory evaluation was performed by a panel of five qualified tea experts from South China Agricultural University. Each panel member held national senior tea assessor certification and possessed over five years of experience in tea descriptive sensory analysis. Infusions were prepared according to the standard method for tea sensory evaluation (GB/T 23776-2018) [28,29]. Specifically, 5 g of each tea sample was infused in a covered teacup with 150 mL of boiling water for exactly 5 min. Panelists then assessed the infusions. Taste and aroma intensity were scored on a scale of 0 to 10, where: 0–2 = very weak, 2–4 = weak, 4–6 = neutral, 6–8 = strong, and 8–10 = extremely strong [30]. Data are expressed as mean values.

### 2.8. Statistical Analysis

Raw data from three biological replicates were preprocessed using Microsoft Excel 2021. Significant differences among treatments were evaluated using one-way ANOVA and multifactor ANOVA in SPSS 24 (SPSS Inc., Chicago, IL, USA). Visualization employed bar plots, stacked plots, radar charts, and heatmaps generated with Origin 2024 (OriginLab Corporation, Northampton, MA, USA). Orthogonal partial least squares-discriminant analysis (OPLS-DA) with variable importance in projection (VIP) was performed in SIMCA 14.1 (Umetrics, Umeå, Sweden). Additional heatmaps were created using TBtools 2.136 (Chengjie Chen, Guangzhou, China).

## 3. Results

### 3.1. Quantification of Caffeine, Catechins, and Theanine in NGBTs

**Figure 2:**
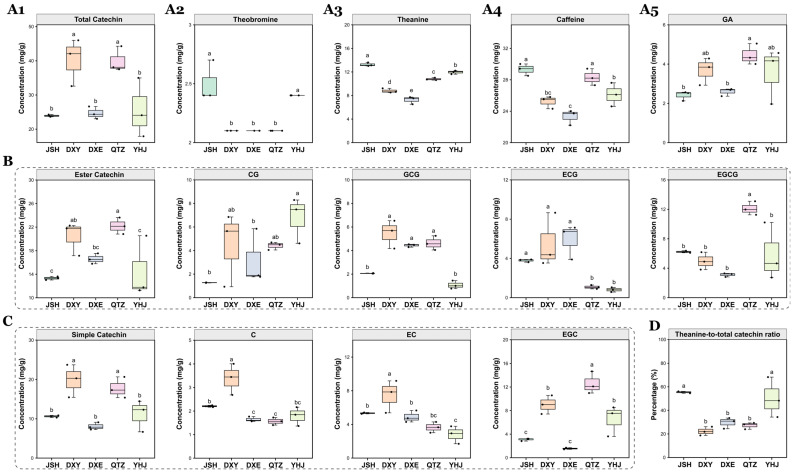
Content of non-volatile compounds in 5 NGBTs. Different letters indicate statistically significant differences according to one-way ANOVA. (**A1**–**A5**) Content of total catechin, theobromine, theanine, caffeine, and GA in 5 NGBTs. (**B**) Content of ester catechin (including CG, GCG, ECG, and EGCG) in 5 NGBTs. (**C**) Content of simple catechin (including C, EC, and EGC) in 5 NGBTs. (**D**) Theanine-to-total catechin ratio in 5 NGBTs.

HPLC was employed to quantify caffeine, total catechins, and theanine in the five NGBTs (Figure 2).

Significant compositional differences were observed among the cultivars. Total catechin content was significantly higher in DXY and QTZ compared to JSH, DXE, and YHJ, though no significant differences were detected between DXY and QTZ or among JSH, DXE, and YHJ. Theobromine levels were significantly elevated in JSH and YHJ relative to DXY, DXE, and QTZ, while no significant differences existed between JSH and YHJ or among DXY, DXE, and QTZ. Theanine content differed significantly across all five teas, with JSH displaying the highest concentration, followed sequentially by YHJ, QTZ, DXY, and DXE. For caffeine, JSH and QTZ exhibited the highest concentrations, followed by YHJ, DXY, and DXE; however, no significant differences were observed between JSH and QTZ, between DXY and YHJ, or between DXY and DXE.

Theanine is widely recognized as a key contributor to the refreshing umami taste (freshness) in tea [31], while catechins are primarily responsible for imparting bitterness and astringency [32]. The ratio of amino acids to catechins serves as a critical biochemical indicator for evaluating the quality attributes of mellowness (thickness) and briskness (freshness) in tea liquors [33]. Notably, JSH and YHJ exhibit significantly higher theanine-to-total catechin (T/C) ratios compared to DXY, DXE, and QTZ (*p* < 0.05).

These pronounced variations in total catechins, theobromine, theanine, caffeine, and gallic acid highlight their potential role as key discriminating non-volatile compounds contributing to the compositional distinctness of the five NGBTs.

**Figure 3:**
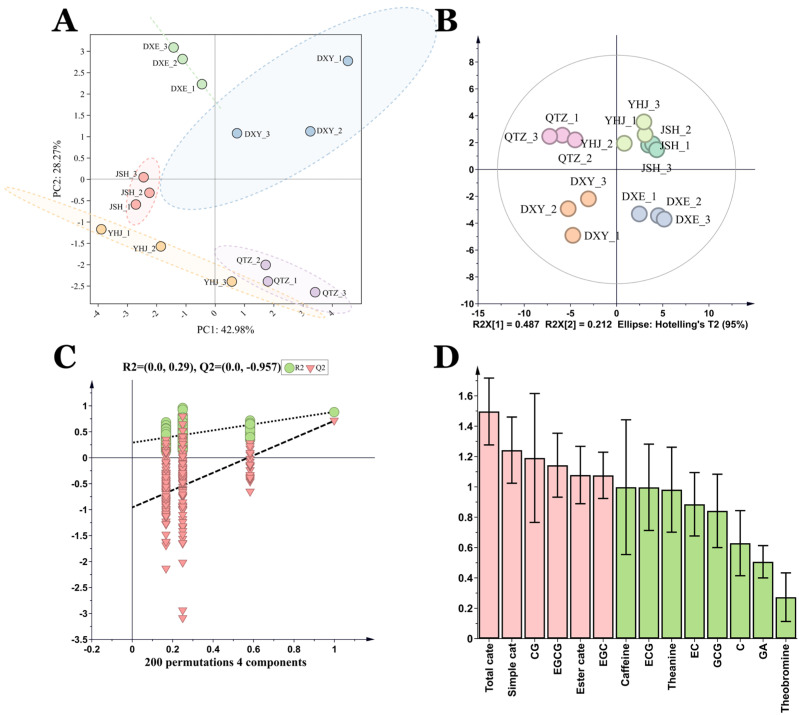
Multivariate statistical analysis of non−volatile components in the 5 NGBTs. (**A**) PCA score plot, the dashed circle represents the 95% confidence range; (**B**) OPLS−DA score plot; (**C**) cross−validation results: the intercept of the Q2 regression line of the cross−validation model with 200 tests of alignment was less than 0, indicating that the OPLS-DA discriminant model was not overfitted and the model was relatively reliable. (**D**) VIP score plot: pink bars represent non-volatile compounds with VIP > 1; green represents VIP < 1.

Principal component analysis (PCA) and orthogonal partial least squares-discriminant analysis (OPLS-DA) were employed to investigate the multivariate relationships among the five NGBT samples based on their non-volatile compound profiles. Both PCA score plots and OPLS-DA score plots demonstrated clear separation among all five NGBTs (Figure 3A,B), confirming significant compositional differences in their non-volatile constituents. The validity and absence of overfitting in the OPLS-DA model were confirmed by permutation testing (Figure 3C). Variable importance in projection (VIP) scores quantified each compound’s contribution to the OPLS-DA model discrimination, with VIP > 1 indicating statistically significant contributions. This analysis identified total catechins, simple catechins, CG, EGCG, esterified catechins, and EGC as possessing VIP scores >1 (Figure 3D), highlighting their potential as key markers differentiating the non-volatile profiles of the five NGBTs.

### 3.2. Volatile Components of NGBTs

#### 3.2.1. Identification and Analysis of Volatile Substances

**Figure 4:**
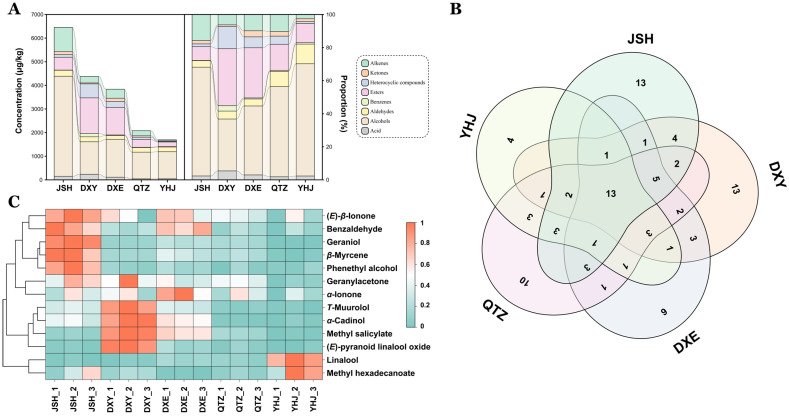
Aroma profile in the 5 NGBTs. (**A**) Volatile substances (left) and proportion (right) stack chart of 5 NGBTs. (**B**) Volatile substance Venn diagram of 5 NGBTs. (**C**) Heat map of 13 common volatile substances.

Volatile compounds in the five NGBTs were profiled using HS-SPME-GC-MS. A total of 97 volatile compounds were identified across chemical classes: acids (3), alcohols (23), aldehydes (9), benzenoids (6), esters (21), heterocyclics (2), ketones (7), and alkenes (26). Total volatile content varied significantly among teas, with JSH exhibiting the highest abundance, followed by DXY, DXE, QTZ, and YHJ. Alcohols and esters constituted the dominant classes across all samples, though their distribution differed markedly: JSH contained the highest alcohol content while YHJ showed the lowest. Notably, JSH displayed the most abundant alkenes profile (Figure 4A), which included multiple aroma-active monoterpenes and sesquiterpenes characteristic of black tea fragrance.

Unique compositional signatures were observed: JSH, DXY, DXE, QTZ, and YHJ contained 13, 13, 6, 10, and 4 unique volatiles, respectively (Figure 4B), against a background of 13 shared aroma compounds. Heatmap analysis of these shared compounds (Figure 4C) revealed distinct enrichment patterns: JSH showed significantly higher relative abundance of (*E*)-*β*-ionone, benzaldehyde, geraniol, *β*-myrcene, and phenethyl alcohol; DXY exhibited elevated *T*-muurolol, *α*-cadinol, methyl salicylate, and (*E*)-pyranoid linalool oxide; DXE had limited enrichment; QTZ displayed uniformly low abundance; and YHJ was characterized by pronounced linalool and methyl hexadecanoate content. This multivariate variability suggests that volatile profiles contribute substantially to the sensory differentiation of NGBTs.

#### 3.2.2. Screening for Volatile Substances

**Figure 5:**
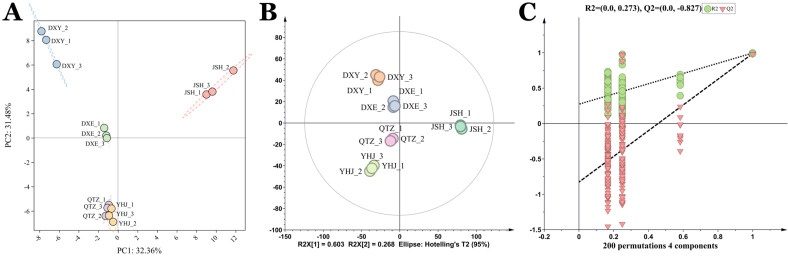
Multivariate statistical analysis of volatile components in the 5 NGBTs. (**A**) PCA score plot, the dashed circle represents the 95% confidence range; (**B**) OPLS−DA score plot; (**C**) cross−validation results: the intercept of the Q2 regression line of the cross-validation model with 200 tests of alignment was less than 0, indicating that the OPLS−DA discriminant model was not overfitted and the model was relatively reliable.

Multivariate analysis using PCA and OPLS-DA was conducted to elucidate volatile compound disparities among the five NGBTs. Both PCA and OPLS-DA score plots revealed clear separation among all samples (Figure 5A,B), confirming significant inter-group differences in volatile profiles. Permutation testing (200 iterations, Q2-intercept = −0.827) validated the OPLS-DA model’s reliability without overfitting (Figure 5C).

**Figure 6:**
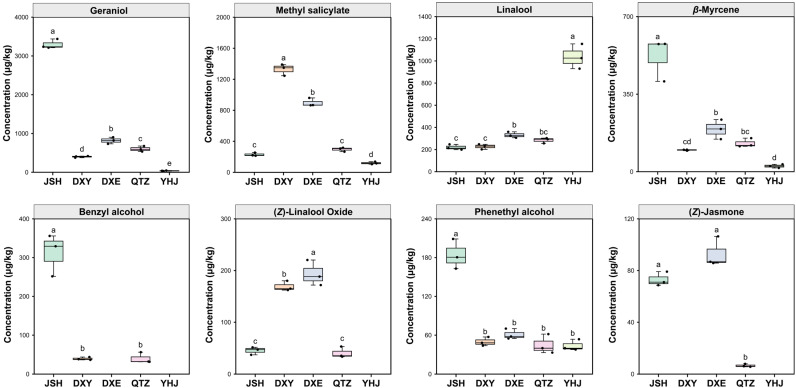
Key compounds with both OAV > 1 and VIP > 1 in the 5 NGBTs, different letters indicate statistically significant differences according to one-way ANOVA.

Fourteen compounds exhibited VIP > 1, identifying them as primary discriminants: Geraniol, Methyl salicylate, Linalool, (*E*)-Geranic acid methyl ester, (*E*)-Pyranoid linalool oxide, *β*-Myrcene, (*E*)-Furan linalool oxide, Phenylacetaldehyde, Benzyl alcohol, (*Z*)-Linalool oxide, Linalool oxide pyranoid, Phenethyl alcohol, Geranic acid, and (*Z*)-Jasmone. Among these, eight compounds demonstrated dual significance by satisfying both VIP > 1 and OAV > 1 criteria (Figure 6 and Figure S1)—namely Geraniol, Methyl salicylate, Linalool, *β*-Myrcene, Benzyl alcohol, (*Z*)-Linalool oxide, Phenethyl alcohol, and (*Z*)-Jasmone—suggesting their joint role as chemical and sensory discriminants (Table 1 and Table 2).

Sample-specific patterns emerged with JSH, exhibiting significantly elevated Geraniol, *β*-Myrcene, Benzyl alcohol, and Phenethyl alcohol (*p* < 0.01 versus other NGBTs), DXY showing dominant Methyl salicylate levels, DXE containing the highest (*Z*)-Linalool oxide, and YHJ characterized by exceptionally high Linalool abundance. These differentially abundant compounds are robust discrimination markers for NGBTs’ authentication and flavor profiling.

### 3.3. Sensory Evaluation of NGBTs

**Figure 7:**
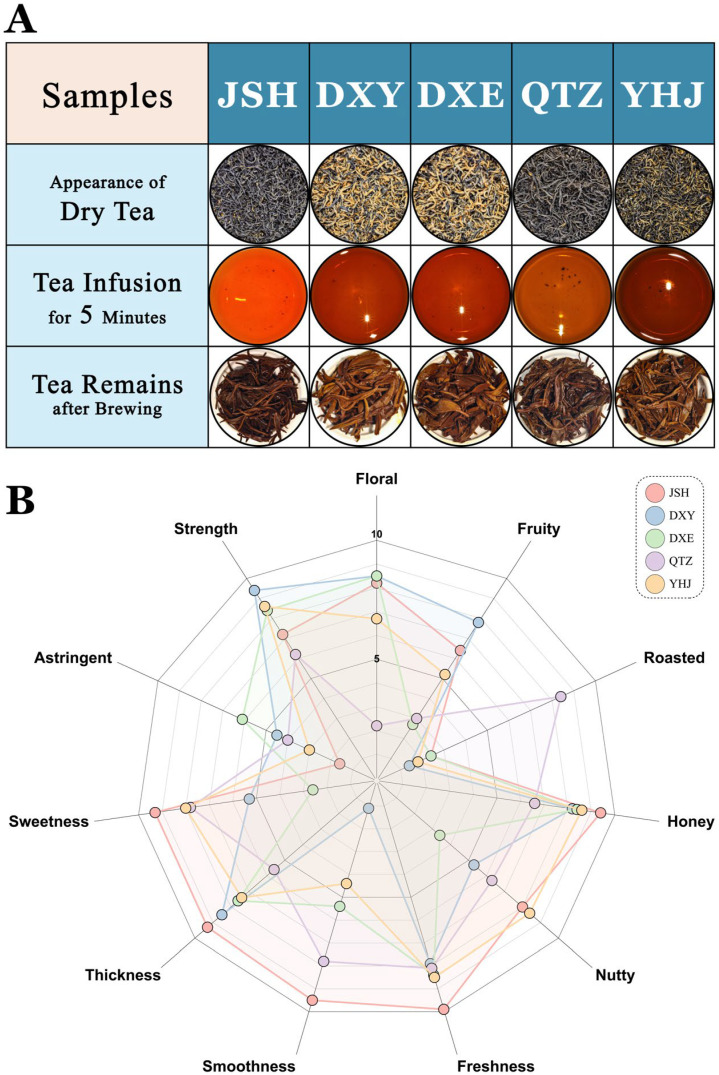
Sensory evaluation of 5 NGBTs. (**A**) Appearance of NGBTs. (**B**) Radar plot based on the results of sensory evaluation in NGBTs.

Sensory evaluation, an effective methodology for investigating tea flavor profiles, was employed to assess the samples. Figure 7A depicts the visual characteristics: JSH exhibited uniformly twisted, slender, and straight dark brown leaves with a glossy appearance and sparse golden tips. DXY and DXE shared similar characteristics, featuring stout, tightly rolled leaves with abundant golden tips. QTZ displayed coarser, dark brown leaves, while YHJ showed uniformly twisted, dark brown, glossy leaves with golden tips. Regarding liquor color, JSH presented the brightest orange-red and most luminous infusion. DXY and DXE produced similar deep red liquors, QTZ showed an intermediate color between JSH and DXY, and YHJ displayed the deepest red hue.

The sensory attribute scores are visualized in the radar chart (Figure 7B). For taste, JSH achieved the highest scores in sweetness, thickness, smoothness, and freshness, coupled with the lowest bitterness, indicating minimal astringency. DXY scored highest in strength and second highest in thickness after JSH. DXE showed relatively high freshness but also exhibited the highest bitterness, revealing a complex taste profile. QTZ demonstrated favorable sweetness and smoothness, second only to JSH. YHJ performed well in strength, following DXY.

Significant variations emerged in the aroma profiles. DXY and DXE exhibited the strongest floral notes, followed by JSH, YHJ, and QTZ. DXY displayed the most pronounced fruity character, trailed by JSH, YHJ, QTZ, and DXE. QTZ dominated the roasted aroma dimension, substantially surpassing the other four samples. JSH possessed the strongest honey-like aroma, while YHJ showed the most distinct nutty notes.

### 3.4. Correlation Analysis Between Sensory Evaluation and the Characteristics of Flavor and Aroma in NGBTs

**Figure 8:**
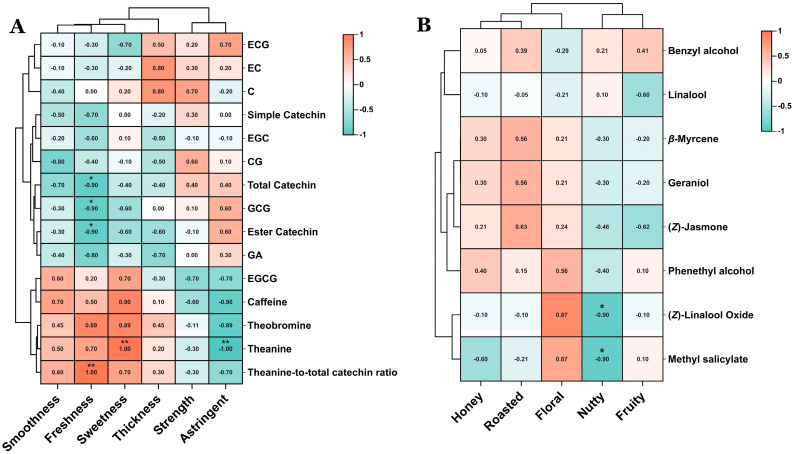
Correlation analysis of sensory evaluation and characteristics of taste and aroma in 5 NGBTs. Statistical significances are denoted by * for *p* < 0.05 and ** for *p* < 0.01. (**A**) Correlation analysis of sensory evaluation and 15 important factors of taste in 5 NGBTs. (**B**) Correlation analysis of sensory evaluation and 8 important volatile compounds of aroma in 5 NGBTs.

Aroma and taste constitute two critical sensory dimensions for evaluating tea quality. To elucidate the relationships between sensory evaluation outcomes and the key flavor attributes of NGBTs, correlation analyses were conducted between the results of sensory evaluation and 12 major non-volatile compounds alongside 8 important volatile compounds (Figure 8A). As shown in the heatmap, total catechins, ester catechins, and GCG exhibited significant negative correlations (*p* < 0.05) with freshness. Conversely, theobromine and the theanine-to-total catechin ratio demonstrated significant positive correlations with freshness. Furthermore, theobromine and theanine showed significant positive correlations with sweetness while displaying significant negative correlations with astringency. Regarding aroma attributes, (*Z*)-Linalool oxide and Methyl salicylate were significantly negatively correlated with nutty notes (Figure 8B). No significant correlations were observed between the remaining compounds and sensory evaluation parameters.

## 4. Discussion

### 4.1. Non-Volatile Signatures and Taste Differentiation

Taste constitutes the most critical quality indicator for NGBT, accounting for 30% of its sensory evaluation score [29,39], with non-volatile compounds serving as primary determinants of taste attributes. Among these, theanine, catechins, and caffeine are established as the most influential factors governing tea taste perception [40,41,42]. Although the five investigated NGBT cultivars share comparable growing environments, genetic differences emerge as the dominant factor driving compositional variation in these compounds [20,34]. Our HPLC quantification revealed significant inter-cultivar differences in theanine, catechin, and caffeine levels, while OPLS-DA modeling identified total catechins, simple catechins, CG, EGCG, esterified catechins, and EGC as discriminative non-volatile markers, establishing a direct linkage between chemical signatures and sensory profiles.

Sensory evaluation confirmed distinct taste characteristics among the cultivars. Theanine, a known sweet-tasting compound, activates umami receptors while suppressing bitterness perception [13,31,43,44]. Notably, JSH and YHJ exhibited significantly elevated theanine content compared to other NGBTs, correlating with their superior sweetness and freshness scores. Conversely, DXE’s low theanine content aligned with its reduced performance in these attributes. Correlation analysis further demonstrated significant positive relationships between theanine and freshness, as well as between theanine and sweetness, while revealing a significant negative correlation with astringency—mechanistically explaining DXE’s pronounced astringency. Catechins, recognized contributors to bitterness and astringency [45], showed an inverse pattern: JSH and YHJ’s lower total catechin levels, combined with negative correlations between total catechins and both sweetness (*p* < 0.05) and freshness (*p* < 0.05), collectively enhanced their sweetness/freshness perception while reducing astringency. While caffeine and theobromine typically impart bitterness that compromises sweetness and freshness [46], their unexpected correlation patterns here likely reflect the combinatorial nature of taste perception, where individual compounds rarely dictate overall quality in isolation.

Critically, the T/F ratio emerged as a fundamental biochemical indicator for taste balance, sharply distinguishing JSH and YHJ from DXY, DXE, and QTZ and explaining the former group’s characteristic fresh-sweet profile. The observed compositional differences are governed by multiple factors: Catechins undergo oxidation into theaflavins/thearubigins during processing [47], with their accumulation further influenced by seasonal variations and bud-to-leaf ratio at harvest. Meanwhile, theanine biosynthesis is regulated by glutamine synthetase, glutamate synthase and glutamate dehydrogenase enzyme activity, and *CsTS1*, *CsAlaDC*, and *CsGS* gene expression [44]. These factors collectively determine the elevated theanine–catechin ratio in JSH and YHJ. Consequently, strategic optimization of processing protocols, harvest season, plucking standards, and cultivar selection is essential for enhancing the taste quality of NGBTs.

### 4.2. Volatile Architecture and Aroma Differentiation

Aroma constitutes a critical quality parameter for NGBT, accounting for 25% of its sensory evaluation score [48,49]. Distinct aroma profiles arise from the combinatorial effects of volatile compounds at varying concentrations [50]. This study identified 97 volatile compounds across chemical classes—including 3 acids, 23 alcohols, 9 aldehydes, 6 benzenoids, 21 esters, 2 heterocyclics, 7 ketones, and 26 alkenes—with significant quantitative differences among the five NGBT cultivars. Critically, compounds exhibiting OAV > 1 and VIP > 1 serve as primary aroma contributors [36,51]. Among the profiled volatiles, 8 dual-criterion markers (OAV > 1 and VIP > 1) were identified as key drivers of cultivar-specific aromas. These markers provide mechanistic insights into the olfactory differentiation of NGBTs and offer actionable targets for optimizing manufacturing processes to align with market preferences.

Geraniol, a monoterpenoid alcohol associated with rose-like floral and honey-sweet notes, is biosynthesized via the methylerythritol phosphate (MEP) pathway and regulated by terpene synthase gene *CsTPS* expression [52,53]. Its significant enrichment in JSH establishes this compound as a signature aroma marker for this cultivar. Conversely, linalool—imparting a citrus-floral character [54,55,56]—dominated YHJ’s volatile profile, consistent with its role in conferring distinctive sensory attributes as reported in prior studies on large-leaf tea cultivars.

Notably, sensory evaluation highlighted YHJ’s pronounced nutty aroma, yet correlation analysis revealed a disconnect: Benzaldehyde—the sole volatile with reported nutty descriptors [57,58]—exhibited low abundance across all cultivars, insufficient to explain YHJ’s high sensory score. This suggests the involvement of non-quantified Maillard reaction products formed during thermal processing, known to generate nutty-roasty notes in black tea.

The aroma composition of NGBTs reflects intricate interactions within terpenoid metabolic networks, particularly monoterpene biosynthesis. Our findings establish a chemical basis for understanding the unique flavor chemistry of NGBTs and provide genomic targets for breeding cultivars optimized for desirable aroma traits.

## 5. Conclusions

This study employed an integrated analytical approach—combining HPLC, GC-MS, OAV assessment, and sensory evaluation with multivariate statistics—to identify key flavor determinants in five NGBT cultivars (JSH, DXY, DXE, QTZ, and YHJ). Significant inter-cultivar taste variations were primarily attributed to differential levels of total catechins, simple catechins, CG, EGCG, esterified catechins, and EGC, as established through compositional analysis and OPLS-DA modeling (VIP > 1). Ninety-seven aroma compounds were identified by GC-MS, with 8 dual-criterion markers (VIP > 1 and OAV ≥ 1) serving as primary discriminants. Notably, geraniol was significantly enriched in JSH, while linalool dominated YHJ’s volatile profile. Sensory assessment confirmed distinct organoleptic signatures: JSH excelled in sweetness, thickness, smoothness, freshness, and honey-like aroma; DXY exhibited pronounced strength, fruity, and floral notes; DXE displayed maximal astringency and floral intensity; QTZ demonstrated superior roasted character; and YHJ was characterized by dominant nutty attributes. Critically, correlation analysis established that the T/F ratio was positively associated with freshness and sweetness (*p* < 0.05). These findings provide a biochemical foundation for understanding the unique flavor chemistry of NGBTs and offer actionable targets for breeding programs aimed at optimizing desirable sensory traits in regional black tea cultivars.

## Figures and Tables

**Table 1:** Volatile compounds with VIP > 1 in OPLS-DA.

No.	Volatile Compound	Odor Type	VIP ^1^	*p*-Value ^2^	OT ^3^ (μg/kg)
1	Geraniol	sweet, floral, fruity, rose, waxy, citrus	5.36	0.000 **	1
2	Methyl salicylate	peppermint, wintergreen mint	3.80	0.000 **	40
3	Linalool	floral, green, fruity	3.76	0.000 **	1
4	(*E*)-Geranic acid methyl ester	n.f.	2.24	0.000 **	n.f.
5	(*E*)-pyranoid linalool oxide	woody	2.15	0.000 **	320
6	*β*-Myrcene	musty, balsamic, spice	2.04	0.000 **	15
7	(*E*)-Furan linalool oxide	flowery	1.94	0.000 **	320
8	Phenylacetaldehyde	floral, honey, rose, cherry	1.79	0.001 **	6.3
9	Benzyl alcohol	floral, rose, phenol, balsamic	1.62	0.000 **	100
10	(*Z*)-Linalool oxide	earthy, floral, sweet, woody	1.58	0.000 **	6
11	Linalool oxide pyranoid	floral, honey	1.29	0.001 **	190
12	Phenethyl alcohol	fruity, rose, sweet, apple	1.25	0.000 **	140
13	Geranic acid	n.f.	1.23	0.002 **	n.f.
14	(*Z*)-Jasmone	woody, herbal, floral, spicy, jasmine, celery	1.17	0.000 **	0.26

^1^ VIP, variable importance in projection. ^2^ ** means *p* < 0.01. ^3^ OT: odor threshold in water; the thresholds for volatile compounds in water mentioned in the table are cited from the references [11,12,25,34,35,36,37,38]; n.f.: not found in the literature.

**Table 2:** Volatile compounds with OAV>1.

No.	Volatile Compound	OAV ^1^				
		JSH	DXY	DXE	QTZ	YHJ
1	Phenethyl alcohol	1.3	0.4	0.4	0.3	0.31
2	*δ*-Cadinene	4.9	18.9	7.5	7.8	-
3	Benzyl alcohol	3.1	0.4	-	0.4	-
4	(*Z*)-Linalool oxide	7.5	28.1	32.2	6.7	-
5	*α*-Ionone	9.0	9.2	13.4	9.0	6.90
6	Phenylacetaldehyde	-	22.0	8.7	20.2	24.31
7	Benzaldehyde	2.1	1.3	1.9	1.2	1.19
8	*β*-Myrcene	34.6	6.5	12.7	8.5	1.63
9	(R)-1-Methyl-5-(1-methylvinyl)cyclohexene	1.8	-	-	-	-
10	(*E,Z*)-Alloocimene	1.9	-	0.6	0.4	-
11	(*Z*)-*β*-Ocimene	7.1	-	-	-	-
12	Methyl salicylate	5.7	33.1	22.4	7.4	2.93
13	Dihydroactinidiolide	-	-	-	2.0	2.05
14	Methyl jasmonate	10.6	6.0	4.3	-	0.40
15	(*E*)-Furan linalool oxide	0.3	1.1	0.6	0.2	-
16	(*Z*)-Jasmone	280.0	-	357.2	24.7	-
17	Limonene	-	-	7.1	2.9	1.37
18	(*E*)-Citral	-	-	1.5	-	-
19	*o*-Cymene	-	6.9	-	-	-
20	(*E*)-β-Ionone	487.6	323.0	398.1	326.8	278.51
21	Nerol	1.1	1.5	-	0.3	0.11
22	Geraniol	3292.5	396.5	814.0	596.5	33.64
23	Hotrienol	-	2.5	-	-	-
24	Nerolidol	-	164.8	62.8	81.9	36.48
25	Linalool	218.6	225.3	329.3	282.7	1035.18
26	Citral	23.2	3.4	-	4.1	-

^1^ OAV, odor activity value. “-” means that the calculation cannot be performed.

## Data Availability

The original contributions presented in the study are included in the article/Supplementary Material, and further inquiries can be directed to the corresponding author.

## Supplementary Materials

The following supporting information can be downloaded at: https://www.mdpi.com/article/10.3390/foods14142466/s1, Table S1: The volatile components of 5 NGBTs; Table S2: The calibration curve information of Caffeine, Theanine, and Catechins; Table S3: The result of sensory evaluation in 5 NGBTs; Table S4: The VIP value in OPLS-DA of non-volatile compounds; Table S5: The VIP value in OPLS-DA of volatile compounds; Figure S1: The VIP value in OPLS-DA of volatile compounds; Figure S2: GC-MS chromatogram of 5 NGBTs; Figure S3: HPLC chromatogram of 5 NGBTs.

## Abbreviations

The following abbreviations are used in this manuscript:

NGBT	Northern Guangdong black tea
HPLC	High-performance liquid chromatography
HS-SPME-GC-MS	Headspace solid-phase microextraction gas chromatography–mass spectrometry
OPLS-DA	Orthogonal partial least squares-discriminant analysis
VIP	Variable importance in projection
PCA	Principal component analysis
OAV	Odor activity value
C	Catechin
EC	Epicatechin
GC	Gallocatechin
EGC	Epigallocatechin
CG	Catechin gallate
ECG	Epigallocatechin
GCG	Gallocatechin gallate
EGCG	Epigallocatechin gallate
RI	Retention index
