# Supplementary material for: Metabolomics and Sensory Evaluation Reveal the Aroma and Taste Profile of Northern Guangdong Black Tea
Source: Foods. 2025 Jul 14;14(14):2466. doi: 10.3390/foods14142466 (PMC12294277; doi:10.3390/foods14142466)
Supplement: Supplementary file 1 [file foods-14-02466-s001.zip › foods-3732399-supplementary.pdf]

Table S1. The volatile components of 5 NGBTs.

No.	Volatile Compounds	Classification	Concentration (μg/kg) [1]					RI(cal)/RI(ref) [2]	ID[3]
			JSH	DXY	DXE	QTZ	YHJ		
1	(*E*)-3-Hexenoic acid	Acid	-	-	-	-	37.96±7.13	1021/1002	MS, RI
2	Geranic acid		143.01±33.94	27.4±1.17	107.91±26.81	36.37±13.04	-	1355/1352	MS, RI
3	(*E*)-Geranic acid		-	204.48±5.24	-	-	-	1355/1363	MS, RI
4	Phenethyl alcohol	Alcohols	183.9±18.91	49.44±5.61	60.73±6.59	44.71±12.09	43.59±6.99	1116/1106	MS, RI
5	Linalool oxide pyranoid		94.18±18.14	-	110.4±21.95	5.52±1.95	-	1178/1163	MS, RI
6	4-isopropylbenzyl alcohol		-	20.99±1.21	-	-	-	1289/1282	MS, RI
7	(*E*)-3-Hexen-1-ol		-	-	-	62.31±0	-	856/855	MS, RI
8	Dihydro-β-ionol		-	-	-	2.64±1.06	-	1449/1434	MS, RI
9	*α*-Cadinol		5.74±1	13.23±1.01	7.98±0.48	0.68±0.06	0.8±0.37	1653/1643	MS, RI
10	Cedrol		-	-	-	0.87±0.21	1.19±0.65	1598/1591	MS, RI
11	Benzyl alcohol		311.89±44.2	39.28±3.13	-	39.79±11.52	-	1036/1028	MS, RI
12	(*Z*)-Linalool oxide		45.01±5.94	168.78±7.9	193.22±20.16	40.44±8.7	-	1074/1064	MS, RI
13	Epicubenol		-	-	-	2.31±0.42	-	1627/1616	MS, RI
14	2-Heptanol		-	-	63.85±5.9	-	-	901/903	MS, RI
15	(*E*)-Nerolidol		26.51±2.53	-	-	-	-	1564/1551	MS, RI
16	*T*-Muurolol		4.85±0.41	11.35±0.77	4.71±0.37	1.53±0.17	1.17±0.2	1642/1630	MS, RI
17	Nerol		54.03±5.43	72.14±5.82	-	12.77±1.76	5.44±0.91	1228/1219	MS, RI
18	Geraniol		3292.46±101.14	396.47±17.61	814.01±69.69	596.46±58.7	33.64±6.42	1255/1250	MS, RI
19	Hotrienol		-	277.01±13.07	-	-	-	1107/1096	MS, RI
20	(*E*)-Carveol		-	35.95±3.23	-	-	-	1217/1210	MS, RI
21	Perillyl alcohol		-	21.79±1.46	-	-	-	1296/1289	MS, RI
22	Nerolidol		-	41.2±2.65	15.7±1.87	20.49±1.23	9.12±3.24	1565/1551	MS, RI
23	*α*-Terpineol		-	-	-	7.54±1.6	13.51±1.39	1190/1181	MS, RI
24	(*E*)-Geraniol		-	-	-	9.98±2.18	-	1260/1252	MS, RI
25	Linalool		218.59±20.08	225.34±19.15	329.33±22.05	282.68±20.17	1035.18±91.56	1099/1092	MS, RI
26	Phytol		-	-	-	-	1.79±0.17	2114/2093	MS, RI
27	Phenylacetaldehyde	Aldehydes	-	138.74±3.9	54.93±7.52	127.11±18.84	153.17±19.81	1045/1035	MS, RI
28	(*E*)-2-Hexenal		51.63±0.3	-	-	-	-	854/854	MS, RI
29	Benzaldehyde		49.81±4.31	31.1±1.26	46.74±2.29	29.78±1.91	28.49±3.22	962/962	MS, RI
30	Cocal		2.55±0.5	-	-	2.56±0.45	4.17±1.21	1486/1479	MS, RI
31	Phellandral		-	16.46±0.88	-	-	-	1276/1266	MS, RI
32	2-phenylbut-2-enal		-	7.06±0.66	17.84±1.36	12.33±2.97	14.32±4.51	1279/1264	MS, RI

Table S1. (continued)

No.	Volatile Compounds	Classification	Concentration (μg/kg) [1]					RI(cal)/RI(ref) [2]	ID[3]
			JSH	DXY	DXE	QTZ	YHJ		
33	(*E*)-Citral	Aldehydes	-	-	42.89±3.36	-	-	1270/1261	MS, RI
34	Neral		33.66±0.84	-	-	-	-	1240/1231	MS, RI
35	Citral		115.85±3.6	16.94±0.55	-	20.47±2.08	-	1276/1263	MS, RI
36	Caryophyllene	Alkenes	3.71±1.2	9.9±3.02	13.82±1.2	3.82±0.24	-	1419/1409	MS, RI
37	*α*-Farnesene		8.01±1.71	-	-	-	-	1508/1495	MS, RI
38	*δ*-Cadinene		7.42±1.39	28.31±6.25	11.31±1.62	11.68±0.42	-	1524/1512	MS, RI
39	*β*-Bisabolene		-	6.2±2.07	-	-	-	1509/1496	MS, RI
40	*γ*-Cadinene		-	5.27±1.59	4.09±0.53	-	-	1513/1503	MS, RI
41	*α*-Calacorene		-	6.64±1.26	3.27±0.56	2.51±0.26	-	1542/1532	MS, RI
42	*α*-Corocalene		-	0.87±0.28	-	-	-	1623/1611	MS, RI
43	Cadalene		-	2.8±0.4	1.75±0.22	0.94±0.15	-	1674/1663	MS, RI
44	*γ*-Elemene		-	-	-	6.54±0.6	-	1433/1422	MS, RI
45	*β*-Farnesene		-	-	-	2.77±0.28	1.94±0.36	1444/1444	MS, RI
46	*γ*-Muurolene		-	-	-	1.45±0.31	-	1477/1462	MS, RI
47	*α*-Amorphene		-	-	5.69±0.62	-	-	1482/1469	MS, RI
48	*β*-Myrcene		519.59±79.56	97.11±2.1	190.94±35.99	127.6±16.45	24.47±6.8	993/994	MS, RI
49	2-Carene		29.89±0.95	42.64±1.4	-	-	-	1001/1009	MS, RI
50	(R)-1-Methyl-5-(1-methylvinyl)cyclohexene		80.04±12.49	-	-	-	-	1027/1023	MS, RI
51	(*E*,*Z*)-Alloocimene		65.32±8.62	-	20.21±1.76	13.18±2.79	-	1131/1125	MS, RI
52	(*Z*)-*β*-Ocimene		242.64±28.69	-	-	-	-	1038/1040	MS, RI
53	(*E*,*E*)-Alloocimene		69.44±8.89	-	-	-	-	1144/1133	MS, RI
54	*γ*-Terpinene		-	43.63±7.9	-	-	-	1060/1048	MS, RI
55	3,4-Dimethyl-2,4,6-octatriene		-	12.68±1.9	31.62±2.2	-	-	1121/1120	MS, RI
56	1,3,8-p-Menthatriene		-	7.16±0.56	-	-	-	1119/1125	MS, RI
57	Alloocimene		-	14.46±2.95	-	10.69±2.76	4.66±1.81	1142/1134	MS, RI
58	Limonene		-	-	70.79±6.54	29.16±1.14	13.75±3.14	1030/1021	MS, RI
59	3-Carene		-	-	27.08±6.73	-	-	1011/1007	MS, RI
60	Theaspirane		-	-	-	4.17±0.21	-	1305/1305	MS, RI
61	Neophytadiene		-	-	0.77±0.15	1.78±0.54	-	1837/1821	MS, RI
62	Naphthalene	Benzenes	-	-	14.39±5.23	-	-	1182/1174	MS, RI
63	Butylated Hydroxytoluene (BHT)		5.83±0.94	9.66±0.89	-	10.69±3.41	14.27±3.71	1513/1499	MS, RI
64	2,4-Di-tert-butylphenol		-	-	9.65±0.86	-	-	1519/1499	MS, RI

Table S1. (continued)

No.	Volatile Compounds	Classification	Concentration (μg/kg) [1]					RI(cal)/RI(ref) [2]	ID[3]
			JSH	DXY	DXE	QTZ	YHJ		
65	*m*-Cymene	Benzenes	-	49.93±9.29	-	-	-	1023/1016	MS, RI
66	*o*-Cymene		-	78.89±8.96	-	-	-	1027/1019	MS, RI
67	Dehydro-ar-ionene		2.17±0.44	3.4±0.25	-	-	-	1354/1343	MS, RI
68	(*E*)-3-Butanoic acid, 3-hexenyl ester	Esters	14.59±1.35	13.03±0.78	-	-	-	1185/1177	MS, RI
69	Methyl salicylate		227.09±18.28	1325.8±60.79	895.24±44.08	294.54±22.16	117.36±13.69	1197/1187	MS, RI
70	(*Z*)-3-Hexenyl hexanoate		100.63±98.25	-	16.27±1.34	18.47±1.44	8.93±2.35	1380/1369	MS, RI
71	Hexyl hexanoate		36.62±9.45	-	-	-	-	1384/1375	MS, RI
72	(*E*)-2-Hexenyl Hexanoate		29.88±7.24	-	-	7.56±0.65	3.35±0.9	1391/1377	MS, RI
73	Phenethyl butyrate		5.93±1.12	-	-	-	-	1447/1430	MS, RI
74	Geranyl butyrate		14.82±1.96	-	-	-	-	1562/1547	MS, RI
75	(*Z*)-3-Hexenyl benzoate		5.69±1.22	5.52±0.31	-	-	-	1570/1559	MS, RI
76	(*Z*)-3-Hexenyl 2-methylbutyrate		-	28.18±2.38	-	-	-	1234/1222	MS, RI
77	Diisobutyl phthalate		-	4.97±1.43	4.47±0.63	-	9.05±3.79	1870/1853	MS, RI
78	Dihydroactinidiolide		-	-	-	4.29±0.42	4.3±1.6	1532/1519	MS, RI
79	Methyl jasmonate		31.8±3.53	18.09±1.48	12.95±1.25	-	1.2±0.33	1638/1636	MS, RI
80	Neryl hexanoate		6.93±1.06	-	-	-	-	1732/1739	MS, RI
81	Methyl hexadecanoate		2.54±0.8	1.91±0.15	1.39±0.03	1.66±0.1	3.73±1.11	1926/1908	MS, RI
82	Geranyl formate		22.06±3.28	-	-	-	-	1300/1292	MS, RI
83	Methyl epijasmonate		-	3.37±0.64	1.68±0.34	-	-	1685/1667	MS, RI
84	Ethyl 2-(5-methyl-5-vinyltetrahydrofuran-2-yl)propan-2-yl carbonate		-	-	-	-	40.64±7.49	1090/1079	MS, RI
85	(*Z*)-3-Hexenyl isovalerate		-	-	-	-	8.73±3.91	1238/1221	MS, RI
86	Geranyl acetate		10.78±5.33	-	-	-	-	1382/1372	MS, RI
87	Dihydroactinidiolide		6.56±1.22	-	-	-	-	1538/1519	MS, RI
88	(*E*)-Geranic acid methyl ester		24.66±2.89	107.16±8.91	227.8±19.14	-	-	1324/1313	MS, RI
89	(*E*)-pyranoid linalool oxide	Heterocyclic compounds	22.67±3.52	247.26±11.06	50.46±10.49	29.61±10	17.65±5	1173/1162	MS, RI
90	(*E*)-Furan linalool oxide		87.8±16.05	341.79±16.4	202.92±24.54	74.26±20.66	-	1086/1079	MS, RI
91	*α*-Ionone	Ketones	3.61±0.72	3.68±0.8	5.34±0.86	3.6±0.78	2.76±0.66	1426/1416	MS, RI
92	(*Z*)-Jasmone		72.81±4.51	-	92.87±9.52	6.43±0.98	-	1394/1389	MS, RI
93	*β*-Damascenone		-	-	-	7.24±0.53	-	1386/1374	MS, RI

Table S1. (continued)

No.	Volatile Compounds	Classification	Concentration (μg/kg) [1]					RI(cal)/RI(ref) [2]	ID[3]
			JSH	DXY	DXE	QTZ	YHJ		
94	Dehydro-*β*-ionone	Ketones	-	-	-	2.1±0.46	-	1485/1472	MS, RI
95	7,9-Di-tert-butyl-1-oxaspiro(4,5)deca-6,9-diene-2,8-dione		-	-	-	1.75±0.52	-	1923/1902	MS, RI
96	Geranylacetone		8.44±1.75	8.77±2.69	6.06±1.16	6.53±0.49	2.55±0.41	1453/1441	MS, RI
97	(*E*)-β-Ionone		43.89±2.32	29.07±7.83	35.83±4.21	29.41±1.72	25.07±6.53	1486/1474	MS, RI

[1] "-" means not detected. Values are shown as mean ± SD (n=3).

[2] RI(cal), retention index obtained using N-alkane calculations. RI(ref), the published retention index of compounds in the NIST 14 library.

[3] ID, Identification method. MS, identification based on the NIST 2014 mass spectral database; RI, retention index.

Table S2. The calibration curve information of caffeine, theanine, and catechins.

Name	RT[1]	Peak Area															Channel	R	R^2	CT[2]	Equation[3]
		JSH			DXY			DXE			QTZ			YHJ							
Theanine	7.228	1168277	1234475	1177806	730312	795616	723001	525029	624051	643106	939164	971229	929937	1091796	1026216	1068835	W2489 ChA	0.994503	0.989036	LC	Y = 9.97e+006 X - 1.27e+005
Theobromine	7.778	332584	327021	317096	218119	220439	208243	241385	242656	236034	244825	225905	231773	295343	281655	264434	W2489 ChA	0.999626	0.999252	LC	Y = 7.18e+007 X - 2.83e+005
Caffeine	10.955	5890577	5777819	5631950	4808850	5069441	5027693	4661623	4714002	4402565	5778992	5401653	5544042	5448847	5177344	4854962	W2489 ChA	0.999952	0.999904	LC	Y = 5.87e+007 X + 4.69e+004
C	14.835	304003	295404	293218	680420	559857	393433	200061	164231	159613	157392	124964	188006	109941	216735	280631	W2489 ChA	0.999984	0.999968	LC	Y = 7.54e+006 X + 2.62e+004
EC	16.111	1057512	1028154	1044541	1840118	1563717	1047166	1110689	916919	827610	690456	563103	825533	284735	541811	717405	W2489 ChA	0.999905	0.99981	LC	Y = 9.23e+006 X - 1.40e+004
ECG	19.785	1564600	1701598	1459152	3868799	1907601	1532506	1698203	3164436	2989899	395725	335246	518715	181467	285327	397740	W2489 ChA	0.999905	0.99981	LC	Y = 9.23e+006 X - 1.40e+004
EGC	13.275	149025	148777	1401345	536926	454253	370212	68575	60446	53939	616055	556867	751382	170713	375307	429450	W2489 ChA	0.999864	0.999729	LC	Y = 2.34e+006 X - 6.21e+003
EGCG	16.492	2155734	2254187	2036972	2179954	1717448	1321870	1130162	1100776	951874	4045935	352325	4712652	1624322	924969	3664789	W2489 ChA	0.999819	0.999637	LC	Y = 1.83e+007 X - 2.04e+005
GCG	17.048	653748	675782	661224	2282108	1987603	1430410	1554239	1475616	1537075	1380559	1164283	1578529	443520	196540	274600	W2489 ChA	0.999997	0.999994	LC	Y = 1.48e+007 X - 1.48e+005

[1] RT, retention time.

[2] CT, correction type.

[3] X is the concentration (Mg/ml) and Y is the peak area.

Table S3. The result of sensory evaluation in 5 NGBTs.

Sample	Floral	Fruity	Roasted	Honey	Nutty	Freshness	Smoothness	Thickness	Sweetness	Astringent	Strength
**JSH**	8.2	6.4	2.4	9.4	8	9.9	9.5	9.3	9.3	1.6	7.2
**DXY**	8.5	7.8	1.4	8.2	5.3	7.9	1.1	8.5	5.3	4.5	9.4
**DXE**	8.5	2.7	2.4	8.4	3.4	8.4	5.4	7.6	2.6	6.1	8.4
**QTZ**	2.2	3	8.4	6.6	6.3	8.1	7.8	5.6	7.8	4	6.2
**YHJ**	6.7	5.2	1.8	8.6	8.4	8.5	4.4	7.4	8	3	8.6

Table S4. The VIP value in OPLS-DA of non-volatile compounds.

Var ID (Primary)	VIP
Total catechin	1.49705
Simple catechin	1.2421
CG	1.19053
EGCG	1.1427
Ester catechin	1.07804
EGC	1.07596

Table S5. The VIP value in OPLS-DA of volatile compounds.

Var ID (Primary)	VIP
Geraniol	5.36338
Methyl salicylate	3.80232
Linalool	3.75851
(*E*)-Geranic acid methyl ester	2.23725
(*E*)-pyranoidlinalool oxide	2.15034
β-Myrcene	2.04079
(*E*)-Furan linalool oxide	1.94469
Phenylacetaldehyde	1.79239
Benzyl alcohol	1.62157
(*Z*)-Linalool Oxide	1.58153
Linalool oxide pyranoid	1.29103
Phenethyl alcohol	1.24871
Geranic acid	1.23113
(*Z*)-Jasmone	1.17163
Geraniol	5.36338

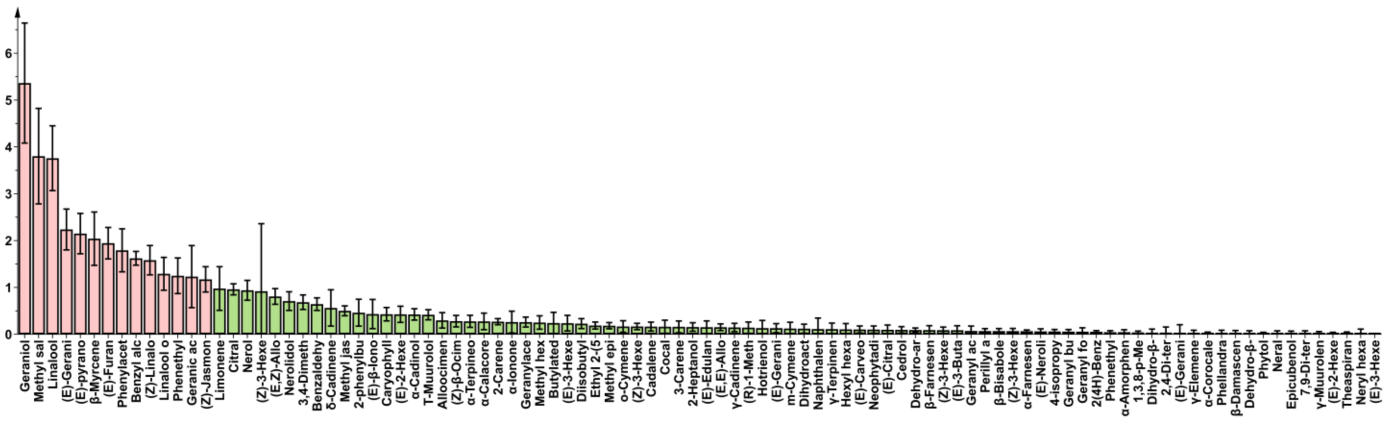

Figure S1. The VIP value in OPLS-DA of volatile compounds. VIP score plot: pink bars represent non-volatile compounds with VIP > 1; green represents VIP < 1.

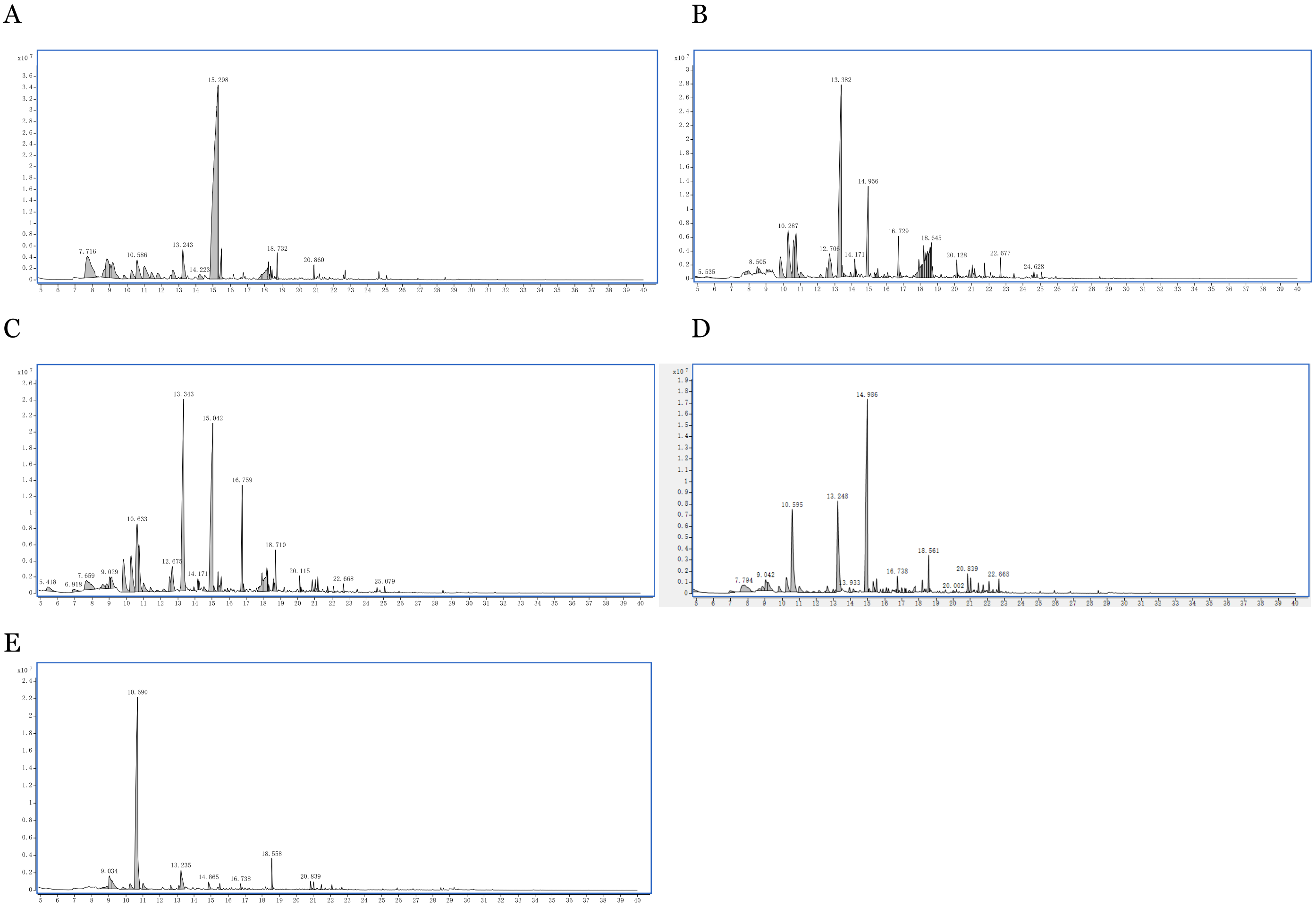

Figure S2. GC-MS chromatogram of 5 NGBTs. (**A-E**) GC-MS chromatogram of JSH (**A**), DXY (**B**), DXE (**C**), YHJ (**D**), and QTZ (**E**).

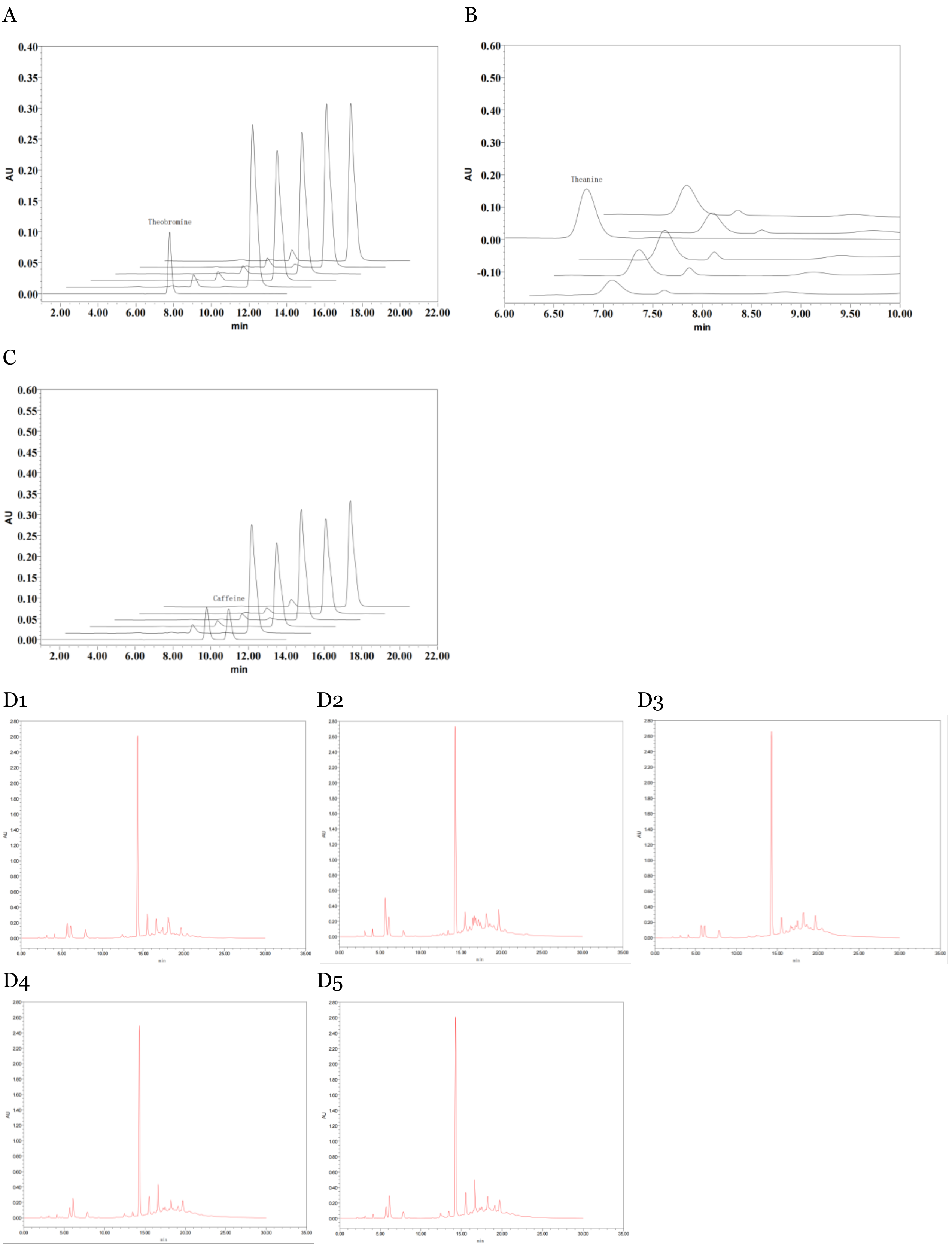

Figure S3. HPLC chromatogram of 5 NGBTs. (**A-C**) HPLC chromatogram of theobromine (**A**), theanine (**B**), and caffeine (**C**) in 5 NGBTs. (**D1-D5**) HPLC chromatogram of catechins in JSH (**D1**), DXY (**D2**), DXE (**D3**), YHJ (**D4**), and QTZ (**D5**).
